# The analysis reveals novel hub genes and pathways associated with Tetralogy of Fallot

**DOI:** 10.3389/fcvm.2025.1604939

**Published:** 2025-11-03

**Authors:** Heling Wen, Zheng Huang, Yujie Mao, Wenjie Tian, Lei Peng, Yu Chen

**Affiliations:** ^1^ Department of Cardiology, Sichuan Academy of Medical Science & Sichuan Provincial People’s Hospital, University of Electronic Science and Technology of China, Chengdu, China; ^2^ Cardiothoracic Disease Center, The Affiliated Tumor Hospital of Chengdu Medical College, Chengdu Seventh People’s Hospital, Chengdu, China; ^3^ Institute of Dermatology and Venereology, Sichuan Provincial People’s Hospital, University of Electronic Science and Technology of China, Chengdu, China; ^4^ Department of Nephrology, Sichuan Academy of Medical Science & Sichuan Provincial People’s Hospital, University of Electronic Science and Technology of China, Chengdu, China

**Keywords:** bioinformatic analysis, TOF, GEO, hub genes, pathway

## Abstract

**Background:**

Tetralogy of Fallot (TOF) is one of the most common complex congenital heart diseases, posing a severe threat to infant health. This study aims to investigate the core genes and pathways associated with TOF to identify potential targets for prevention and intervention. This study aims to explore the core genes and pathways associated with TOF, providing a reference for identifying potential targets for the prevention and intervention of this disease.

**Methods:**

Bulk and single-cell RNA-seq datasets were employed in this study. Differentially expressed genes (DEGs) were calculated, and functional enrichment analysis was performed. The expression of the hub genes was validated by quantitative real-time polymerase chain reaction (qRT-PCR). The dysregulated genes and pathways were further validated at the single-cell resolution.

**Results:**

The DEGs analyzed from two datasets, GSE146220 and GSE217772, were subjected to intersection analysis, resulting in 29 consistently upregulated genes (UpGs) and 22 consistently downregulated genes (DpGs). The qRT-PCR analysis confirmed that the expression of upregulated and downregulated genes was generally consistent with the results of the bioinformatics analysis. Functional enrichment analysis based on four distinct databases has shown that fatty-acid beta-oxidation and related pathways were consistently enriched. With snRNA-seq data, we found that the UpGs were enriched in cardiomyocytes, and the DpGs were enriched in cardiofibroblasts. Meanwhile, it was shown that the TOF tissue had a higher ssGSEA score for fatty acid metabolism-associated pathways. Notably, these fatty acid metabolism pathways were mostly enriched in cardiomyocytes.

**Conclusion:**

This study identified that TOF DEGs are highly enriched in fatty-acid beta-oxidation and related pathways. The snRNA-seq data of TOF showed that fatty acid metabolism pathways are predominantly enriched in cardiomyocytes. These findings contribute to further understanding the potential pathogenic mechanisms of TOF and identifying potential therapeutic targets.

## Introduction

Congenital heart disease refers to a range of structural and functional abnormalities in the heart and major blood vessels that arise from disturbances during embryonic development. A study incorporating global data estimated the prevalence of congenital heart disease, including mild lesions such as ventricular septal defects, atrial septal defects, and patent ductus arteriosus, to be 8–10 per 1,000 live births (1). Congenital heart disease accounts for 28% of all birth defects worldwide (2).

Tetralogy of Fallot (TOF) is the most common cyanotic heart disease, with an incidence rate of about 1 in 30,000 births, accounting for 5% of all congenital heart diseases (3). If not diagnosed and treated in time, TOF can lead to secondary cardiac enlargement and heart failure, ultimately resulting in death. The mortality rate is high; within 10 years, the mortality rate of untreated patients can be as high as 70%–75%, making it the most common congenital heart disease requiring early intervention (4). Therefore, exploring the pathogenesis of TOF holds significant importance for the prevention and treatment of this disease.

Extensive human studies and animal model research have demonstrated that genetic factors play a crucial role in the development of TOF (5, 6). The study reveals that a wide range of genetic abnormalities can contribute to the occurrence of this condition. These abnormalities include chromosomal anomalies such as aneuploidy and structural mutations, as well as rare single nucleotide variants that affect specific genes (7). In mammals, the heart develops from two mesodermal heart fields, the First Heart Field (FHF) and the Second Heart Field (SHF), with FHF cells differentiating first to form the left ventricle, while SHF cells later contribute to the right ventricular outflow tract (OFT) and other structures (8, 9). The OFT is a structure formed during heart development, connecting the ventricles to the arteries, and serves as an important channel for blood to flow from the heart to the body and lungs. In TOF, outflow tract abnormalities are common pathological features. The entire process of cardiac development, from primitive cardiac cells to the mature heart, involves a series of complex signaling pathways and transcriptional coordination (10). Studies suggest that TOF development is linked to disruptions in processes like second heart field progenitor cell deployment, embryonic left/right signaling, and OFT cushion formation, reflecting complex intercellular signaling events (11). In the process of cardiac development, various biological processes, such as abnormal migration of neural crest cells, and functional abnormalities in cardiomyocytes and endothelial cells, may contribute to its formation (12, 13). However, our understanding of the signaling pathways and molecular mechanisms involved in TOF remains insufficient.

To gain deeper insights into the complex molecular mechanisms behind TOF, we adopted a comprehensive bioinformatics approach. This approach involves integrating various data, including bulk and snRNA-seq data, to reveal the complex interactions of various factors at the molecular level. This holistic view not only deepens our understanding of the pathophysiology of the disease but also paves the way for the development of more targeted and effective therapeutic interventions (14). Various bioinformatics analysis methods can be carried out based on gene expression profiles, including studies on differentially expressed genes (DEGs), creating Venn diagrams, studying functional and pathway enrichment, and analyzing protein-protein interactions (PPI) networks.

## Materials and methods

### Data collection

The transcriptomic datasets were retrieved from the Gene Expression Omnibus (https://www.ncbi.nlm.nih.gov/geo/, including GSE203274 for single-nucleic RNA-seq, GSE146220 for a microarray dataset, and GSE217772 for bulk RNA-seq). GSE203274 includes the Fastq form of single-nucleic RNA-seq data based on Illumina Novaseq 6000 platform (15). Six cardiac tissues of TOF (SRR19266878, SRR19266879, SRR19266880, SRR19266881, SRR19266882, SRR19266883) and three tissue of normal (SRR19266894, SRR19266896, SRR19266898) were utilized in our study. Two bulk expression profiles were also utilized. GSE146220 is a microarray dataset containing five heart tissues of patients diagnosed as Tetralogy of Fallot and four from control. The study has not yet been published. GSE217772 is a bulk RNA-seq dataset based on Illumina NovaSeq 6000. This dataset includes five cardiac tissues of patient with Tetralogy of Fallot and five from heathy control, with the study not being published neither.

### SnRNA-seq data processing

The raw data was retrieved and transformed into fastq files using SRA toolkit. These datasets were further transformed to count matrixes using the Cellranger sofware (v7.0.1) based on homo sapiens reference genome GRCh38. The Seurat framework was subsequently employed for the downstream analysis (16). Briefly, a minimum of 500 genes and 35% mitochondrial was set as the cutoff for the removement of low-quality cells because cardiomyocytes contain more mitochondria. The batch effects were eliminated using the Harmony algorithm (17). Harmony achieves data iteration by clustering similar cells from different batches while maximizing the heterogeneity of batches within each cluster. In each iteration, Harmony computes a correction factor for individual cells and applies it to the data, effectively mitigating batch effects while preserving inherent biological variations. Doublets were eliminated using the R package “DoubletFinder” (17). Data preprocessing was based on cell cycle genes and known technical noise features. Then, DoubletFinder simulated dual cell populations to generate artificial double cells, which were then compared with the actual data to identify and eliminate the double cell effect. This approach significantly enhanced the accuracy and reliability of our dataset. The scaled data with top 3,000 variable genes were used to perform principal component analysis (PCA). The first 15 principal components were used for further clustering. To reduce the dimensionality of snRNA-seq data, we employed the Uniform Manifold Approximation and Projection (UMAP) technique. UMAP is a renowned method for dimensionality reduction that effectively preserves both global and local structures of the data in a lower-dimensional space. Marker genes of each type of cell were curated in previous study (15). Differentially expressed genes (DEGs) for each cell cluster between TOF and normal samples were identified using the “FindMarkers” function (Wilcoxon rank-sum test) within the Seurat package, following aggregation into “pseudobulk” samples to enhance statistical power. Genes were considered differentially expressed if they exhibited an |log2FC| > 0.25 and an FDR-adjusted *p*-value <0.1. Pseudobulk analysis was conducted by aggregating 50 cells within a cluster to form a pseudobulk sample. The total count of a gene was used as gene expression. Expression density of specific genes was exhibited using the R package “Nebulosa” (18). Single sample gene set enrichment analysis (ssGSEA) was conducted to score the expression of genes at single-cell resolution.

### Bulk expression matrix processing and data analysis

The microarray expression matrix was log transformed for further analysis. DEGs were identified from the microarray dataset (GSE146220) using the “limma” R package (19) and from the RNA-seq dataset (GSE217772) using the “edgeR” R package (20). For both analyses, genes with an absolute log2 fold change (|log2FC|) >1 and a false discovery rate (FDR) adjusted *p*-value <0.05 were considered statistically significant. The complete lists of DEGs from both datasets have been included as Supplementary Tables S1, S2. Functional enrichment analysis was performed using the webtool Metascape (https://metascape.org/gp/index.html) (21). The protein-protein interaction (PPI) network was constructed using the STRING web tool (v11.0). Only interactions with a combined score >0.7 (high confidence) were included in the network. Disconnected nodes were hidden from the final network visualization.

### Quantitative real-time polymerase chain reaction (qRT-PCR)

The samples used for qRT-PCR analysis were obtained from the outflow tract portion of fetal TOF heart tissues, collected from women who voluntarily terminated their pregnancies during the second trimester, a critical period for diagnosing congenital heart defects by ultrasound. TOF fetuses with other developmental abnormalities were excluded. Normal control outflow tract samples were obtained from second-trimester therapeutic terminations for severe maternal health reasons. Echocardiography confirmed that these fetuses had normal cardiac development and no other developmental defects. The Medical Ethics Committee of Sichuan Provincial People's Hospital approved the study protocol and the collection of human samples, and written informed consent was obtained from the parents of each participant (No. 016292). All experiments complied with the Helsinki Declaration and relevant national laws.

Total RNA was extracted from heart tissues using TRIzol reagent (Life Technologies, Carlsbad, CA, USA). Reverse transcription of the RNA was performed using a high-capacity cDNA reverse transcription kit (OSAKA, JAPAN). Quantitative real-time PCR (qRT-PCR) was carried out with SYBR Green PCR Master Mix (Roche, Germany) according to the manufacturer's instructions, on a BIO-GENER Real-Time System (China). Each sample was run in triplicate. The cycle threshold (CT) values were normalized to glyceraldehyde-3-phosphate dehydrogenase (GAPDH) measured on the same plate, and the 2^−*ΔΔ*CT^ method was used to calculate fold differences in gene expression (22). We selected genes with high node degree in the PPI network for validation in the human TOF group and normal control group. The upregulated genes (UpGs) selected include *UQCRC1*, *UQCRQ*, *HADHA*, and *HADHB*. The downregulated genes (DpGs) selected include *COL5A1*, *COL1A2*, *THBS2*, *TAGLN*, and *ELN*. The primer sequences for the target genes and reference genes have been provided in a table format within the supplementary materials (Supplementary Table S3).

### Statistics

All the statistics was performed using the R software (v4.3.1). Wilcoxon test was employed for the calculation of DEGs of each cluster of snRNA-seq data. The *p*-value was adjusted by False Discovery Rate (FDR). FDR < 0.1 was considered statistically significant in DEGs identification and enrichment analysis unless otherwise specified.

## Results

### Fatty acid metabolism was involved in TOF

**Figure 1:**
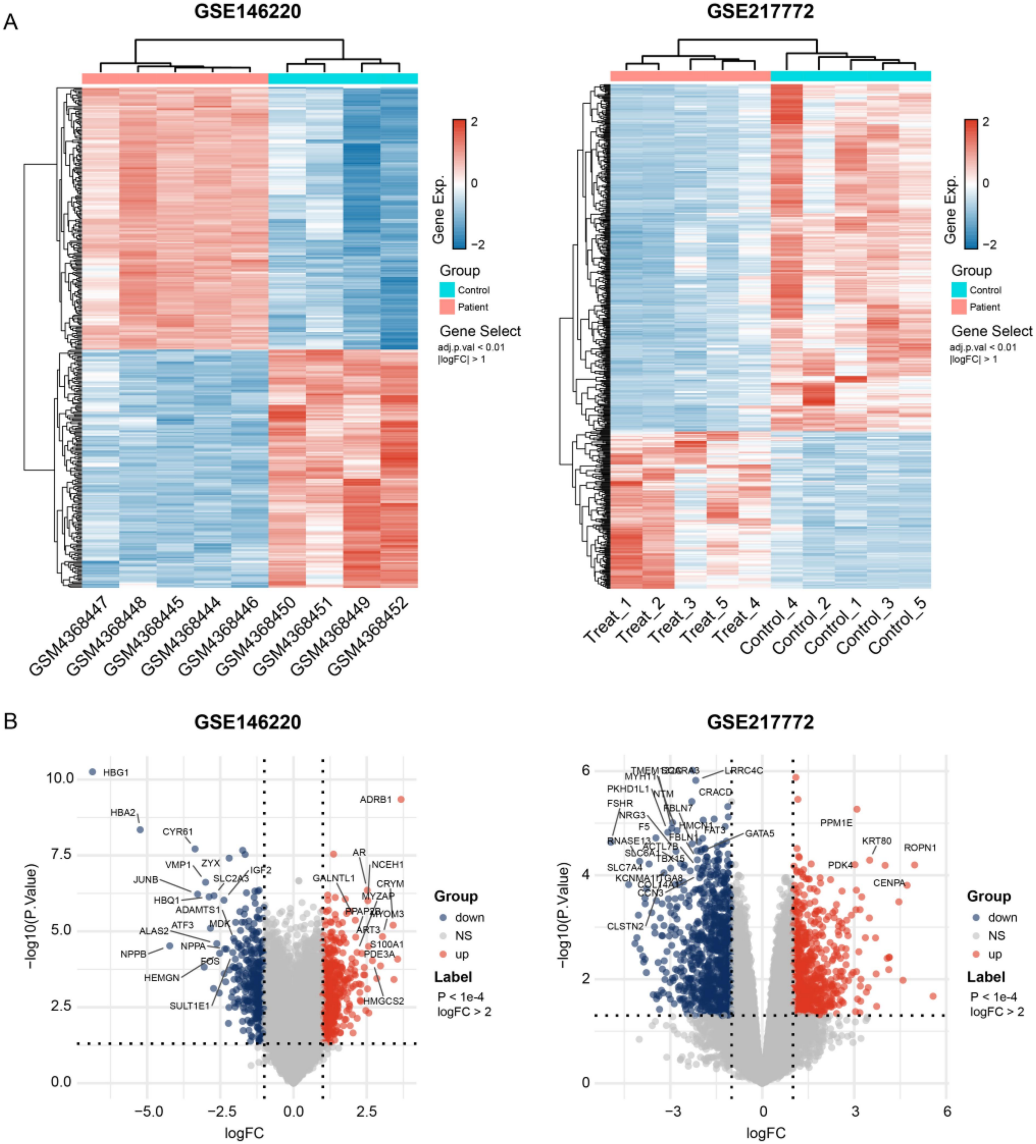
Gene expression dysregulation of the tralogy of fallot. **(A)** Heatmap shows the DEGs between TOF and control samples in two independent datasets. **(B)** Volcano plots exhibit the up- and down-regulated genes in each dataset. The *p*-values shown are adjusted for multiple comparisons using the False Discovery Rate (FDR) method.

**Figure 2:**
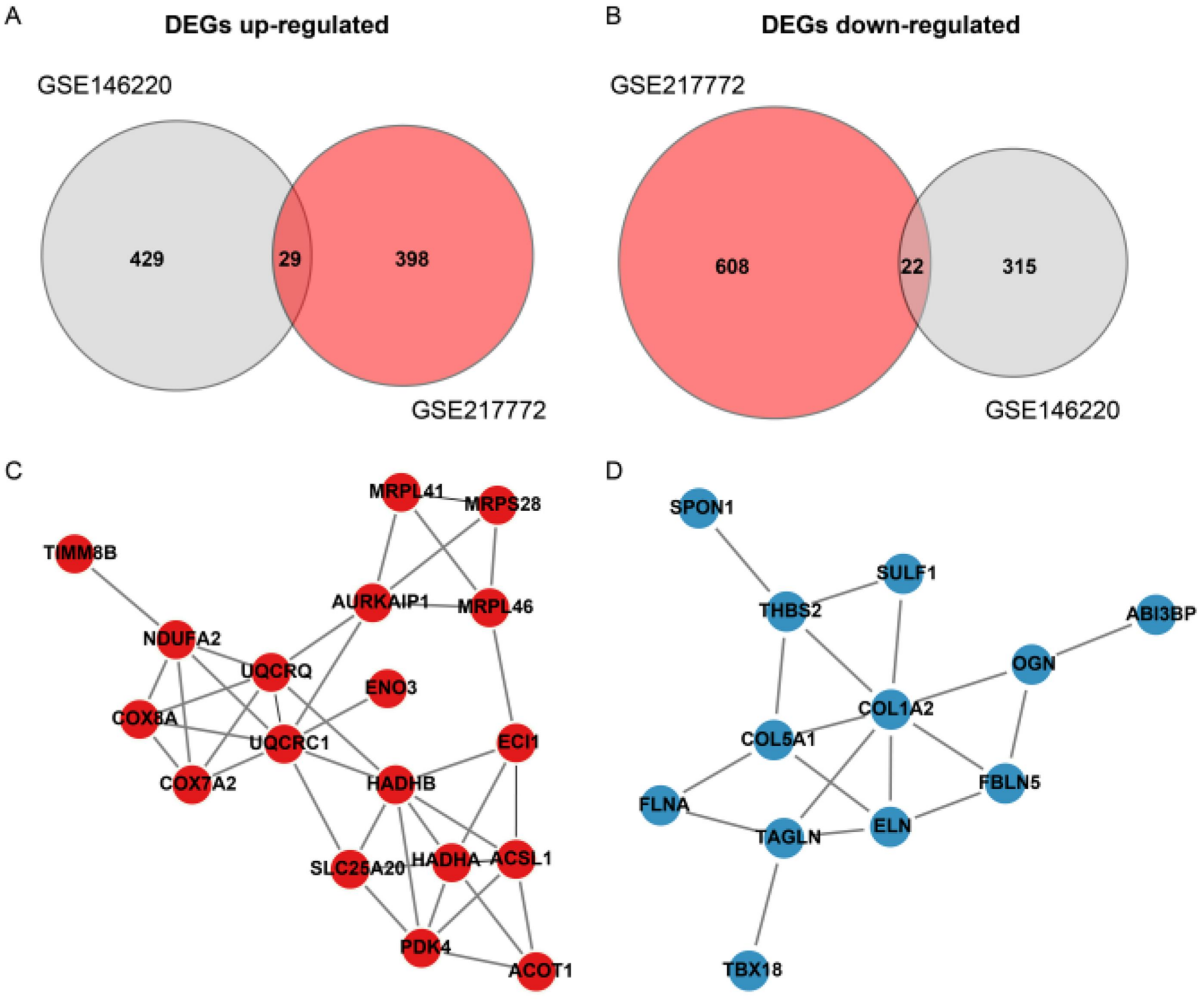
Protein-Protein interaction (PPI) network of differentially expressed genes (DEGs) in TOF. **(A,B)** Venn diagrams illustrate the intersection of up-regulated (UpGs) and down-regulated (DpGs) genes from two independent datasets (GSE146220 and GSE217772), identifying 29 consistent UpGs and 22 consistent DpGs in TOF samples. **(C,D)** PPI networks constructed using the STRING database highlight key hub genes among UpGs (e.g., *UQCRC1*, *UQCRQ*, *HADHA*, *HADHB*) and DpGs (e.g., *COL1A2*, *COL5A1*). Nodes represent genes, edges represent functional associations, and disassociated genes were filtered out. Hub genes are defined by high connectivity within the network, implicating their central roles in mitochondrial complex assembly, fatty acid β-oxidation, and extracellular matrix organization.

**Figure 3:**
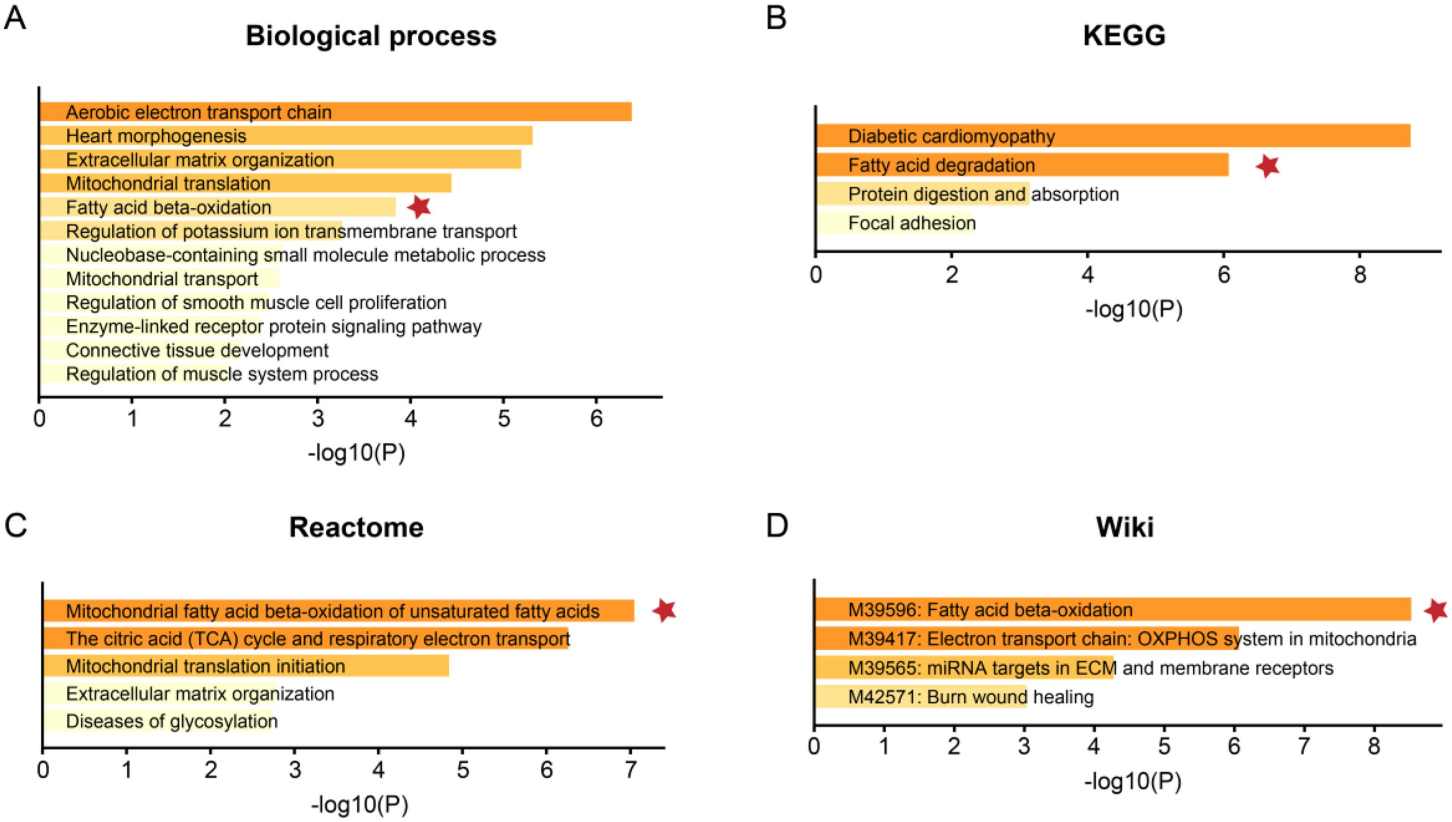
Functional enrichment analysis of the DEGs. The functional enrichment analysis was performed based on the webtool Metascape based on **(A)** biological process, **(B)** the Kyoto Encyclopedia of Genes and Genomes (KEGG), **(C)** Reactome, and **(D)** Wiki pathway databases. The color gradient of the bars represents the statistical significance of the enrichment, quantified as -log10(FDR-adjusted *p*-value). A more intense color indicates a higher level of significance.

The bulk data sets of cardiac tissue of TOF and healthy control were first leveraged to explore genes potentially participated in disease progression. DEGs were calculated and exhibited (Figure 1A). Describing the association between genes labeled out in Figure 1B with TOF-related signaling pathways (Figure 1B). The consistent DEGs in two cohorts were intersected, resulting in 29 consistent UpGs (Figure 2B) and 22 DpGs (Figure 2B). were constructed, indicating intense interactions between these genes. We used the STRING network tool to exclude genes that did not have significant interactions with other genes and constructed the PPI networks. In the constructed PPI network, UpGs such as *UQCRQ*, *UQCRC1*, *HADHA*, and *HADHB* are identified as hub genes, meaning they have the most interaction nodes (Figure 2C). These genes are involved in components of mitochondrial respiratory chain complex III *(UQCRQ*, *UQCRC1*) and key enzymes in the fatty acid β-oxidation pathway (*HADHA*, *HADHB*). In the constructed PPI network of DpGs, genes such as *COL1A2* and *COL5A1* are identified as hub genes, meaning they have the highest number of interaction nodes (Figure 2D). These genes are involved in collagen biosynthesis and extracellular matrix (ECM) remodeling. Functional enrichment analysis was performed to delineate the functions of consistent up- or down-regulated genes based four distinct databases. As a result, these DEGs were top enriched in biological process including aerobic electron transport chain, heart morphogenesis, and extracellular matrix organization, indicating an implication in TOF (Figure 3A). The KEGG enrichment analysis of DEGs highlighted significant involvement in pathways such as diabetic cardiomyopathy, fatty acid degradation, protein digestion and absorption, and focal adhesion (Figure 3B). The Reactome enrichment analysis of DEGs revealed significant involvement in pathways such as mitochondrial fatty acid beta-oxidation of unsaturated fatty acids, the citrate (TCA) cycle and respiratory electron transport, mitochondrial translation initiation, and others (Figure 3C). The Wiki enrichment analysis of DEGs highlighted significant involvement in pathways such as fatty acid β-oxidation, the electron transport chain (OXPHOS system in mitochondria), etc. (Figure 3D). Interestingly, fatty-acid beta-oxidation and related pathways were consistently enriched, indicating an association between fatty acid metabolism and TOF.

### snRNA-seq findings

**Figure 4:**
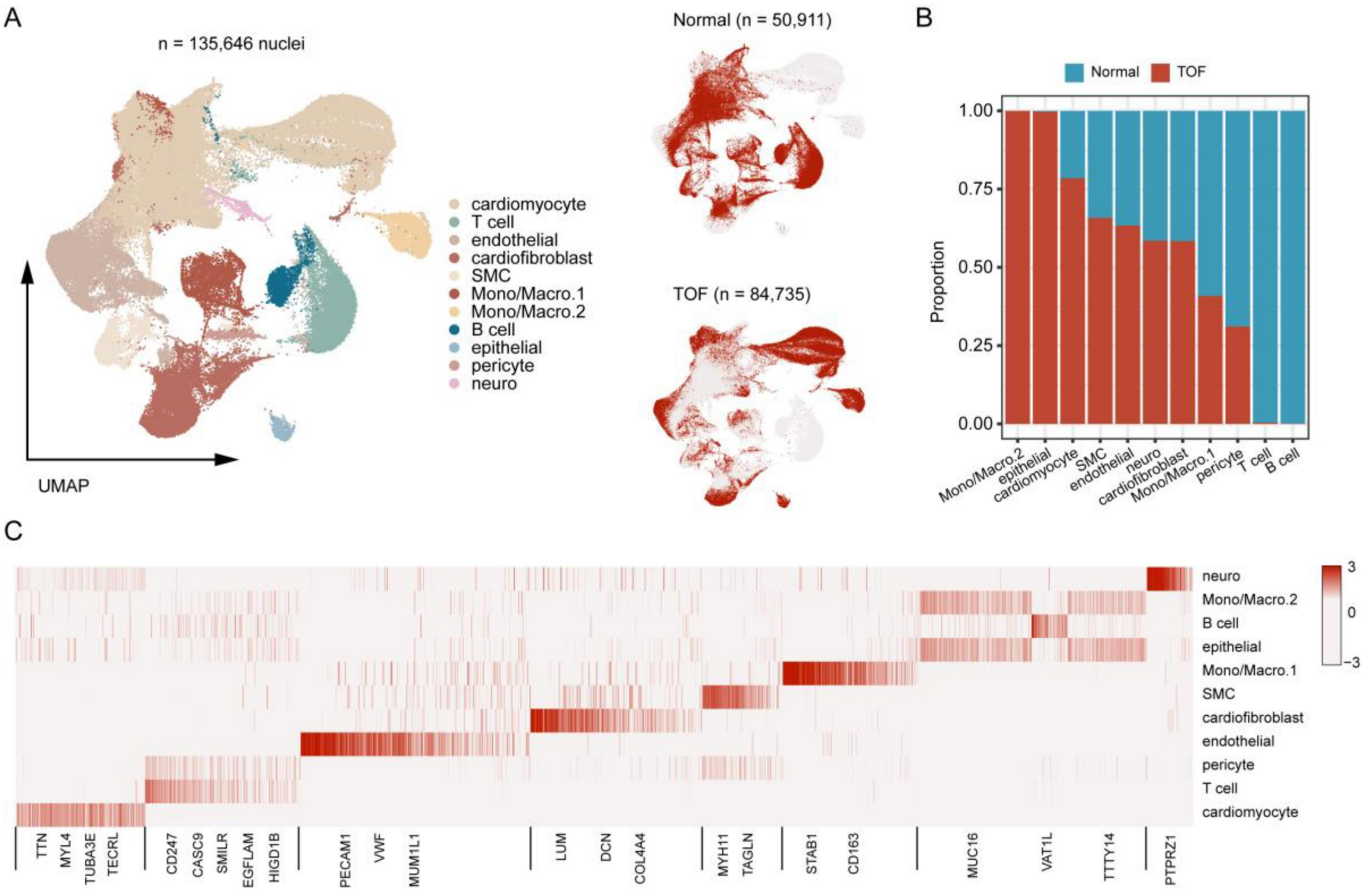
Single-Nucleus RNA sequencing (snRNA-Seq) atlas of TOF and normal heart tissues. **(A)** UMAP projection of integrated snRNA-Seq data from 6 TOF and 3 normal heart tissues, color-coded by cell type. A total of 11 distinct cell populations were identified. **(B)** Bar plot showing the proportional distribution of cell types in TOF vs. normal tissues, revealing increased proportions of monocytes/macrophages, epithelial-like cells, cardiomyocytes, smooth muscle cells (SMCs), endothelial cells, and cardiofibroblasts in TOF. **(C)** Heatmap displaying marker gene expression for each cell cluster, used to annotate cell identities. Rows represent genes, columns represent cell clusters, and color intensity indicates normalized expression levels. The heatmap displays the expression of key canonical marker genes used for the annotation of each cell cluster. The complete list of markers is available in Supplementary Table S4.

**Figure 5:**
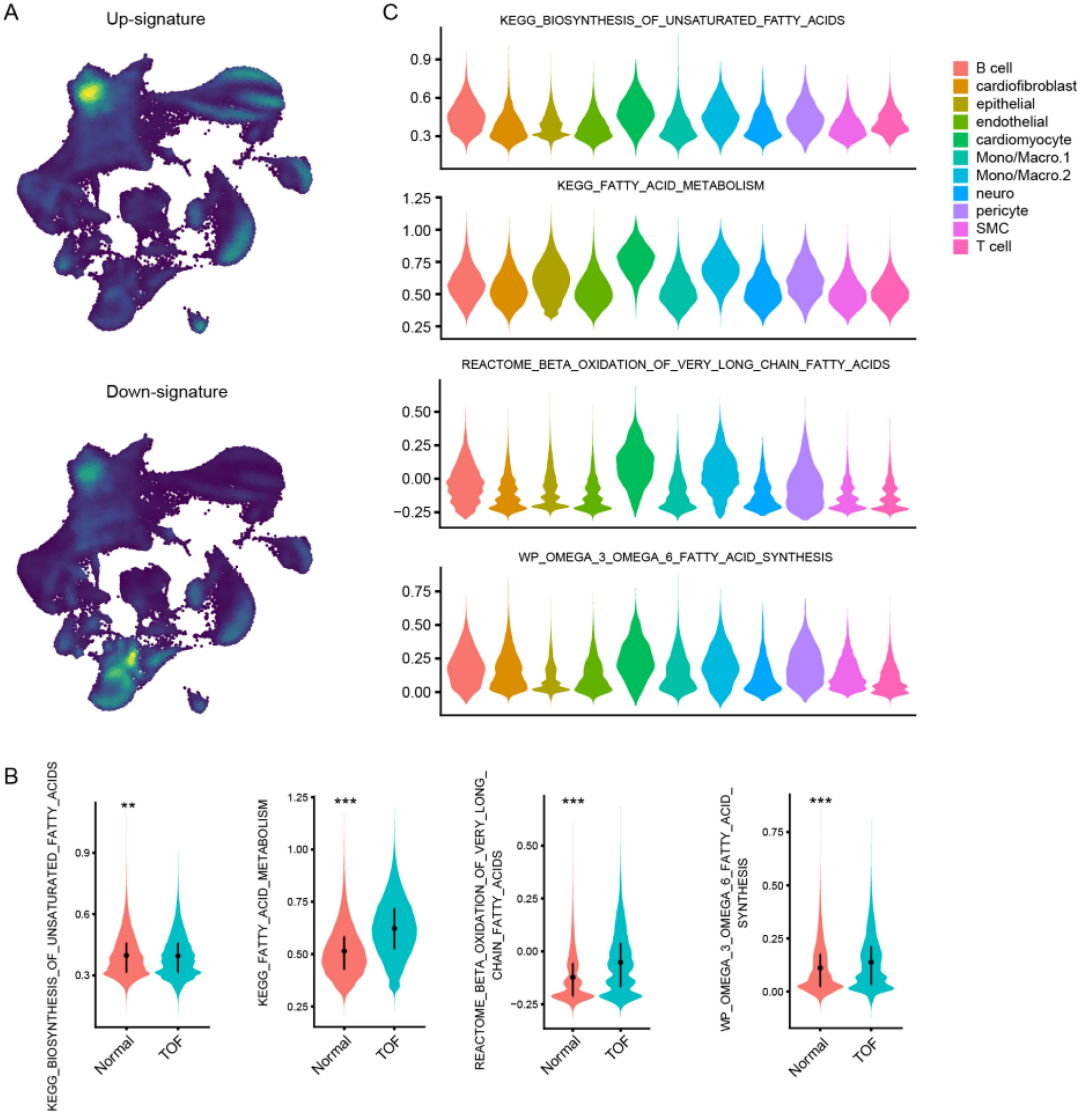
Single-Cell resolution analysis of fatty acid metabolism dysregulation in TOF. **(A)** UMAP visualization showing spatial enrichment of bulk-derived UpGs (enriched in cardiomyocytes) and DpGs (enriched in cardiofibroblasts) within the snRNA-Seq dataset. **(B)** Violin plots comparing ssGSEA scores of fatty acid metabolism-related pathways between TOF and normal tissues. TOF tissues show significantly elevated activity in pathways including fatty acid metabolism, very long-chain fatty acid β-oxidation, and omega-3/omega-6 fatty acid synthesis. **(C)** Cellular decomposition of ssGSEA scores reveals that cardiomyocytes are the primary contributors to the enrichment of fatty acid metabolic pathways in TOF.

To further explore the molecular underpinnings of TOF at single-cell resolution, expression profiles of six cardiac tissues of TOF and three normal sample underwent snRNA-seq were collected. Stringent quality control procedures were conducted, including Harmony-based batch effect elimination, DoubletFinder-based doublet detection, providing a reliable foundation for subsequent analysis (Supplementary Figure S1). As a result, a total of 135,646 quantified cells were identified, with 50,911 from normal and 84,735 from TOF (Figures 4A–C). Unbiased clustering identified 11 types of cells, including cardiomyocyte, endothelial cell (endothelial), smooth muscle cells (SMC), cardiofibroblast, epithelial-like cell, pericyte, monocyte/macrophage, lymphocyte, and a small amount of neuron (Figure 4A). The proportion of different cell subpopulations in the cardiac tissue of the two sample groups differs. Compared to the normal control group, the TOF group shows an increased proportion of monocytes/macrophages, epithelial-like cells, cardiomyocytes, smooth muscle cells (SMCs), endothelial cells, and others in the cardiac tissue (Figure 4B). The heatmap displaying differentially expressed genes of each cell type highlights marker genes used to distinguish between various cell subpopulations (Figure 4C). We have defined up-regulated and DpGs that may be involved in the pathology of TOF. UMAP embeddings disclosed that the UpGs were enriched in cardiomyocytes, and the DpGs were enriched in cardiofibroblast (Figure 5A). The TOF tissue exhibited significantly elevated ssGSEA scores in fatty acid metabolism-associated pathways compared to normal controls. Specifically, these pathways included fatty acid metabolism, beta oxidation of very long chain fatty acids, and omega-3 and omega-6 fatty acids synthesis (Figure 5B). These findings collectively indicate that TOF tissue possesses a markedly higher level of fatty acid metabolic activity. Notably, our pathway enrichment analysis revealed that these fatty acid metabolic pathways were predominantly active within cardiomyocytes (Figure 5C). This cell-specific enrichment suggests that dysregulated fatty acid metabolism in cardiomyocytes may contribute significantly to the pathological mechanisms underlying TOF, potentially driving disease progression through impaired energy homeostasis or lipotoxic effects.

### Validation of hub genes

**Figure 6:**
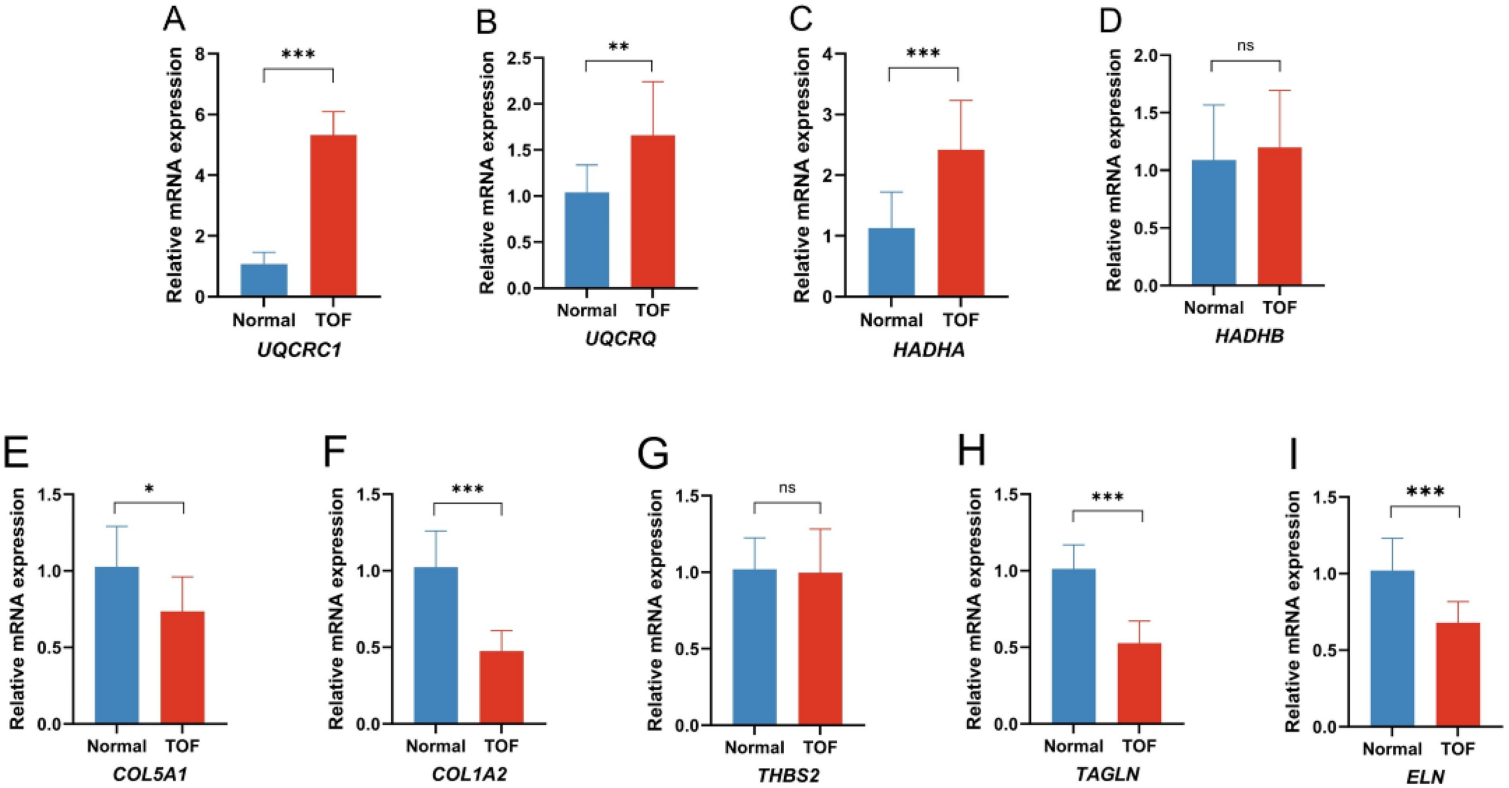
Quantitative real-time PCR of the differential expression genes (DEGs) in the TOF patients compared to the control. **(A)** Expression of *UQCRC1* between the two groups; **(B)** expression of *UQCRQ* between the two groups; **(C)** expression of *HADHA* between the two groups; **(D)** expression of *HADHB* between the two groups; **(E)** expression of *COL5A1* between the two groups; **(F)** expression of *COL1A2* between the two groups; **(G)** expression of *THBS2* between the two groups; **(H)** expression of *TAGLN* between the two groups; **(I)** expression of *ELN* between the two groups. (*p < 0.05 TOF vs. normal, **p < 0.01 TOF vs. normal, ***p < 0.001 TOF vs. normal).

To confirm the findings from the bioinformatics analyses, qRT-PCR was used to assess the expression of the hub genes in normal and TOF heart tissues. In line with the bioinformatics predictions, TOF heart tissues showed an increased expression of *UQCRC1* (Figure 6A), *UQCRQ* (Figure 6B), and *HADHA* (Figure 6C), along with a decreased expression of *COL5A1* (Figure 6E), *COL1A2* (Figure 6F), *TAGLN* (Figure 6H), and *ELN* (Figure 6I) compared to normal tissues. The expression of *HADHB* (Figure 6D) and *THBS2* (Figure 6G) showed no difference between the two groups.

## Discussion

Congenital heart disease is the most common birth defect, accounting for nearly one-third of all major birth defects (23, 24). Among these, TOF is a common cause of congenital heart defects in newborns, representing one-tenth of all congenital heart lesions (25). Although genetic factors are thought to have a key role in TOF, the signaling pathways and molecular mechanisms involved in TOF remain poorly understood (7, 26). In order to gain a deeper understanding of the molecular mechanisms of the disease, we studied the genetic data of patients with TOF, containing microarray data from the GEO database and data from the bulk RNA-seq dataset on TOF.

We analyzed one bulk RNA-seq dataset and one microarray dataset from the GEO database, including samples from TOF patients and control groups. We identified DEGs from TOF heart tissue samples of GSE146220 and GSE217772 datasets and compared them with healthy samples from the same datasets. Through cross-filtering, we identified 29 consistently UpGs and 22 DpGs. Furthermore, we constructed a PPI network to study the interactions among DEGs, revealing strong interactions among genes. The PPI network shows upregulated genes with key nodes like *UQCRC1/Q* and *MRPL41*, indicating their central roles and multiple interactions within the network. Notably, the upregulated gene *UQCRC1/Q* plays a crucial role in maintaining cardiac energy supply, regulating fatty acid metabolism, and resisting oxidative stress (27). UQCRC1 plays a crucial role in maintaining mitochondrial function. It is an essential component of mitochondrial complex III, which is involved in oxidative phosphorylation and electron transport. In mice, *UQCRC1* knockout led to reduced exercise tolerance, increased apoptosis, altered cardiac structure, and mitochondrial abnormalities after exhaustive exercise (28). The *UQCRQ* gene encodes the ubiquinol-cytochrome c reductase, complex III subunit VII, 9.5 kDa, which is a component of mitochondrial complex III. This complex plays a crucial role in the electron transport chain, a critical pathway for cellular energy production (29). The PPI network also highlights downregulated genes with key nodes, such as *COL1A2*, *COL5A1*, and *ELN*, indicating their central roles and multiple interactions within the network. *COL1A2* and *COL5A1* encode the α2 chain of type I collagen and the α1 chain of type V collagen, respectively, and play important roles in maintaining cardiac structure (30). We validated genes with high node degree in the PPI network in the human TOF group and normal control group, confirming increased expression of *UQCRC1*, *UQCRQ*, and *HADHA* in human TOF, and decreased expression of *COL5A1*, *COL1A2*, *TAGLN*, and *ELN* in human TOF. This is consistent with the results from our bioinformatics analysis.

Using four different databases, we conducted functional enrichment analysis and found that upregulated or downregulated genes are most enriched in biological processes such as the mitochondrial electron transport chain, cardiac morphogenesis, and extracellular matrix organization, particularly highlighting the association of the mitochondrial electron transport chain with fatty acid metabolism. KEGG pathway analysis revealed that DEGs are primarily involved in the fatty acid metabolism pathway; Reactome pathway analysis indicated their involvement in the mitochondrial tricarboxylic acid cycle, electron transport chain, and β-oxidation of fatty acids; while Wiki analysis showed enrichment in fatty acid β-oxidation and aerobic transport chain. These findings suggest that fatty acid metabolism and its related pathways play a crucial role in TOF. As vital nutrients, fatty acids are important energy providers for the human heart and are particularly important for the maintenance of cardiac activity (31, 32).

Research indicates that during cardiomyocyte development, cells undergo a transition from anaerobic glycolysis to oxidative metabolism. In this process, fatty acid β-oxidation is significantly enhanced in cardiomyocytes, providing the necessary energy to support their development (33). Our study found that fatty acid β-oxidation and its associated pathways are enriched in TOF, suggesting that these pathways may be involved in the occurrence and development of TOF.

To delve deeper into the pathological relationship between fatty acid metabolism and TOF, we analyzed snRNA-seq expression profiles from 6 TOF cardiac tissues and 3 normal samples, followed by rigorous downstream analysis within the Seurat framework. After stringent data quality control and deduplication processes, we quantified a total of 135,646 cells, with 50,911 originating from normal tissues and 84,735 from TOF tissues. Subsequently, we conducted a proportion analysis of different sample cells, revealing a significant increase in megakaryocytes, epithelial cells, cardiomyocytes, smooth muscle cells, endothelial cells, nerve cells, and cardiac fibroblasts in TOF tissues, which may be associated with the reshaping of cardiac tissue structure and function.

By performing cross-filtering analysis of DEGs, we defined potential upregulated and downregulated gene features related to TOF pathology. UMAP embedding showed enrichment of upregulated gene features in cardiomyocytes, while downregulated gene features were enriched in cardiac fibroblasts. Specifically, upregulated genes were primarily associated with energy metabolism in cardiac cells, especially fatty acid metabolism, whereas downregulated genes were mainly associated with collagen and tissue proteins.

In the ssGSEA analysis of fatty acid metabolism-related pathways, we observed higher scores in the fatty acid metabolism pathway in TOF tissues, including fatty acid metabolism, beta-oxidation of very-long-chain fatty acids, and the synthesis of ω-3 and ω-6 fatty acids, indicating increased activity of myocardial fatty acid metabolism. The abnormal metabolism of fatty acids has also been confirmed in other studies on TOF (34). These pathways were predominantly enriched in cardiomyocytes, highlighting the pathological role of dysregulated fatty acid metabolism in TOF.

## Conclusion

Our study provides new insights into the molecular mechanisms underlying TOF. By employing comprehensive bioinformatics analyses, we identified key differentially expressed genes and pathways, notably those involved in fatty acid metabolism, which were significantly enriched in TOF tissues. The single-cell RNA sequencing further revealed that dysregulated fatty acid metabolism in cardiomyocytes may play a crucial pathological role in TOF. These findings enhance our understanding of TOF pathogenesis and may contribute to the development of targeted therapeutic interventions.

## Data Availability

The raw data supporting the conclusions of this article will be made available by the authors, without undue reservation.
